# Developing and Testing a Chatbot to Integrate HIV Education Into Family Planning Clinic Waiting Areas in Lusaka, Zambia

**DOI:** 10.9745/GHSP-D-21-00721

**Published:** 2022-10-31

**Authors:** Eileen A. Yam, Edith Namukonda, Tracy McClair, Samir Souidi, Nachela Chelwa, Nelly Muntalima, Michael Mbizvo, Ben Bellows

**Affiliations:** a Population Council, Washington, DC, USA.; b Population Council, Lusaka, Zambia.; c International Rescue Committee, New York, NY, USA.; d Nivi, Inc., Washington, DC, USA.

## Abstract

**Background:**

To maximize protection against both unintended pregnancy and HIV, it is important that family planning (FP) services integrate HIV counseling, both to support method choice and identify potential HIV services of interest, such as pre-exposure prophylaxis (PrEP). However, FP providers often lack sufficient time and knowledge to address HIV vulnerability with clients. To potentially offload some of the initial HIV counseling burden from FP providers, we developed and tested a chatbot that provided information about HIV and dual protection to FP clients in waiting areas of FP clinics in Lusaka, Zambia.

**Chatbot Development:**

We drafted a scripted conversation and tested it in English in formative workshops with Zambian women between the ages of 15 and 49 years. After translating the content to Bemba and Nyanja, we conducted a second round of workshops to validate the translations, before uploading the content into the chatbot platform.

**Chatbot User Test:**

Thirty volunteers tested the chatbot in 3 Lusaka FP clinics, completing an exit survey to provide feedback. A large majority (83%) said they learned new HIV information from the chatbot. Twenty (67%) learned about PrEP for the first time through the chat. Most (96%) reported discussing HIV with the provider, after engaging with the chatbot. In response to an open-ended question, several testers volunteered that they wanted to learn more about PrEP.

**Conclusions:**

Pre-consultation waiting-area time is an underutilized opportunity to impart HIV information to FP clients, thereby preparing them to discuss their dual HIV and pregnancy prevention needs when they see their providers. FP clients expressed particular interest in learning more about PrEP, underscoring the importance of integrating HIV into FP services.

## Abstract (precis)

In response to the need to integrate HIV prevention content with FP counseling, a chatbot was developed and tested for use among FP clients in clinic waiting areas to leverage the time while they wait to see providers and guide them through a digital conversation on preventing both pregnancy and HIV.

## Teaser

**Key Findings:**

- There remains a notable gap in decision aids that support family planning (FP) clients to consider how HIV vulnerability may influence contraceptive method choice. Calls for integrating HIV prevention information into FP services have been limited by several factors, including providers’ lack of time for counseling.
- A chatbot can help alleviate provider burden and leverage the underutilized pre-consultation time while clients wait to see providers. Chatbots can guide FP clients through a digital conversation on preventing both HIV and pregnancy and conveying information on HIV.
- Among FP clients in Lusaka, Zambia, who tested a waiting-area chatbot, most reported learning new HIV information, such as pre-exposure prophylaxis, and nearly all reported subsequently discussing HIV content that they learned from the chatbot with the FP provider.

**Key Implications:**

- Time spent waiting to see providers can be used to prepare FP clients to think about HIV vulnerability and how it may affect their contraceptive method choice.
- Chatbots that provide relevant health information in waiting areas are feasible and promising in urban low- and middle-income country settings.

## BACKGROUND

Across health specialties, patients confront many decisions about their health care, including making critical choices about diagnostic procedures, preventive strategies, and treatment options.^1^ The importance of making informed health care decisions is particularly salient among women seeking family planning (FP) services. In light of the notable proliferation of new contraceptive methods in recent decades—the World Health Organization’s *Family Planning: A Global Handbook for Providers*^2^ includes 20 methods—women often are challenged to weigh countless factors in selecting the contraceptive method that is best for them. Methods may be more or less desirable depending on their individual circumstances, values, and preferences. For instance, women’s contraceptive decisions are influenced by mechanistic product attributes (e.g., mode of administration, frequency of use), method effects (e.g., contraceptive efficacy, side effects, effect duration), social or cultural factors (e.g., partner support, religious values), or practical considerations such as cost or geographic availability of services.^3^

Scholars and practitioners of FP service provision promote patient-centered, shared decision making—with providers contributing clinical knowledge and patients expressing their needs, values, and preferences—as essential to optimizing contraceptive satisfaction and continuation.^4^^,^^5^ To facilitate such shared decision making, an array of decision support tools and job aids have been developed to standardize and systematize patient-centered contraceptive decision making among FP clients.^6^ In addition, in the global health community, there is growing interest in advancing self-care interventions—such as digital educational tools and self-administered diagnostics—to support patients to take an active role in health care decisions, with or without the support of a provider.^7^

However, there remains a notable gap in FP decision aids that explicitly support FP clients to contemplate whether and how HIV vulnerability may influence method choice, particularly in low- and middle-income countries (LMICs). Contraceptive decision making is often complicated by the fact that women seeking to prevent pregnancy also may need protection from HIV and other sexually transmitted infections (STIs). Since the most efficacious contraceptive methods do not confer protection against HIV/STIs, ideally, women take their HIV vulnerability into account when making contraceptive decisions. Condoms protect against both disease and pregnancy, but condoms’ contraceptive efficacy is far lower than that of other modern methods such as injectables or intrauterine devices. However, the most efficacious methods at preventing pregnancy—all of which are non-barrier methods—do not protect against HIV/STIs.^2^ FP clients need to be sensitized to these trade-offs when making their contraceptive decisions. To that end, for 3 decades, there have been calls to integrate HIV and FP services, both to inform method choice and to facilitate providing onward HIV-related services (such as testing) to those who want them.^8^^–^^12^ Furthermore, in LMIC settings where FP providers’ time is limited in the face of high client volume, it is often challenging for them to address HIV vulnerability in a fulsome manner during consultations. FP providers also may feel uncomfortable proactively broaching the stigmatized, sensitive behavioral and interpersonal aspects of HIV risk, such as multiple partnerships or intimate partner violence.

In 2019, interest in FP/HIV integration was revived in light of findings from the Evidence for Contraceptive Options and HIV Outcomes (ECHO) Study, which aimed to compare HIV acquisition risk among users of intramuscular depot medroxyprogesterone acetate, a copper intrauterine device, and a levonorgestrel implant among 7,830 FP clients in sub-Saharan Africa. Although the study team found no substantial difference in HIV risk among the methods evaluated—all of which were highly acceptable and efficacious at preventing pregnancy—overall HIV incidence was high, at 3.81 per 100 woman-years.^13^ This high-profile study’s finding of high HIV acquisition among women seeking pregnancy prevention led to calls for more aggressive integration of HIV prevention strategies—such as oral pre-exposure prophylaxis (PrEP)—into FP services.

The 2019 ECHO Study’s finding of high HIV acquisition among women seeking pregnancy prevention led to calls for more aggressive integration of HIV prevention strategies such as PrEP into FP services.

In response to the need for strengthened HIV prevention content in FP counseling, the Population Council developed and tested a digital HIV/FP intervention intended for use among FP clients in waiting areas of clinics. To leverage this underutilized pre-consultation time^14^ when FP clients are “captive audiences,” we created a prototype of a web-based chatbot that engages users in a digital conversation about HIV and FP, including content about PrEP and dual protection, for instance. A chatbot is a software application that simulates having a conversation with a human, consisting of a series of text exchanges, by which users selected responses to scripted questions, with automated content generated in response to their answers. In recent years, chatbots have increasingly been used to discreetly engage users and effect behavior change across health areas.^15^^–^^17^ With support from the Bill & Melinda Gates Foundation, under the Advancing Integrated HIV/FP Counseling with Evidence (ADVICE) project, we developed an Internet-based HIV/FP chatbot and conducted user testing among FP clients in the waiting areas of 3 public clinics in Lusaka, Zambia—which, as a country with high HIV prevalence and high unmet FP need, was 1 of the countries included in the ECHO Study.^13^

We describe the process for developing the chatbot content and platform, which consisted of (1) conducting a desk review to identify relevant domains of HIV vulnerability to be addressed in the chat, (2) developing content and scripting the chat in English, followed by iterative translation and testing in Bemba and Nyanja, and (3) building the 3-language chat in Microsoft Azure. We also report on quantitative findings from a user test among illustrative users in Lusaka FP clinics. User testing is a phase of the design process for digital tools in which testers try out the interface and functions of, for example, a website, mobile application, or digital service. While user testing methods often are similar to those of public health or implementation science research, user testing typically is much faster, uses smaller sample sizes, and aims to inform product design.^18^

## PROCESS FOR DEVELOPING WAITING-AREA CHATBOT

Development of the chatbot consisted of a scoping review to identify domains of HIV vulnerability to be addressed in the chatbot, followed by the development of the chat “script” (i.e., content), and building of the chatbot. Each step of the development process was highly iterative, consisting of multiple cycles of testing, translation, and refinement.

### Desk Review to Identify Relevant Domains of HIV Vulnerability

The chatbot aimed to engage FP clients in a digital conversation that would facilitate reflection about how their HIV vulnerability may influence their method choice and help identify potential HIV services (such as testing) that may be of interest. To ensure that the chatbot content was appropriately tailored for these purposes, we first conducted a desk review to identify potential HIV topics to include in the conversation. Specifically, we sought to synthesize the individual and interpersonal factors that can increase FP clients’ risk of HIV acquisition. Our review included articles and tools from scientific literature databases (i.e., PubMed, Web of Science, and Scopus), World Health Organization guidance, and the PrEPWatch website (www.prepwatch.org) maintained by AVAC. For literature databases, we used the following search terms: HIV risk, HIV vulnerability, tool, assessment, screening, and index. We included screening tools and articles dated from 2014 to 2020, available in English, and used in any geographic area, any setting (i.e., community or facility), and with any cadre of health care worker. We reviewed 35 unique HIV vulnerability assessment tools, 9 of which were described in academic literature, and 26 of which were identified in gray literature or through expert interviews.^19^ For each tool or article included in our review, we collated the screening questions in a spreadsheet, organized by domains of HIV vulnerability that encompassed both individual and interpersonal characteristics. To narrow the digital conversation to HIV vulnerability domains that would be particularly pertinent to informing FP clients’ contraceptive choice, we selected a subset of the following topics to incorporate into the chatbot content: STI history, alcohol or drug use, inconsistent condom use, use of dual protection, prior HIV testing, >1 sex partner, partner has other sex partners, partner living with HIV, treatment status of partner living with HIV, negotiating power with partner (e.g., condom negotiation), and recent experience of gender-based violence.

The chatbot aimed to engage FP clients in a digital conversation that would facilitate reflection about how their HIV vulnerability may influence method choice and help identify potential HIV services that may be of interest.

Next, we drafted text messages covering these topics in the chat content development phase. The process of writing, validating, and translating these conversational exchanges and building the chatbot is described below.

### Chat Content Development, Validation, and Translation

Once we identified the specific individual and interpersonal characteristics associated with HIV vulnerability that were most pertinent to FP counseling (i.e., sexual behavioral risk and partner characteristics), we drafted the messages that would comprise the content of the digital chat. We aimed to support FP clients to inform themselves about HIV, contemplate whether and how their HIV vulnerability may influence their method choice, and consider other HIV services that may be of interest. To that end, we incorporated into the conversation basic information about HIV and about dual protection (i.e., strategies for protecting oneself against both unintended pregnancy and disease). We first developed draft chat content in English, scripted with a conversational tone, that guided the user through various messages and closed-ended questions, which broadly covered 4 topics: background on HIV, sexual behavioral risk, partner characteristics, and dual protection (Table 1). We aimed to produce a digital conversation that would take less than 20–30 minutes to complete.

**TABLE 1.** Illustrative HIV/FP Chatbot Messages

**Topic**	**Illustrative Chat Question**	**Illustrative Response**
Background on HIV	As you may know, HIV affects many women. Have you heard of HIV?	*[If client responds “No” or “Not sure”]* Let me give you some background on HIV. HIV can be acquired through bodily fluids, including through sexual contact, and through breast milk and blood. HIV destroys the immune system, making it difficult for the body to fight off infection.
Sexual behavioral risk	Male and female condoms are the only contraceptive methods that also protect you from HIV or other STIs. But sometimes it's hard to use condoms every time you have sex. With your partner(s), do you feel like you can always use condoms when you have sex?	*[If client responds “Yes”]* Great! Consistent condom use is an effective way of lowering your chance of getting HIV.
Partner characteristics	Women whose partner(s) has/have HIV could be at risk of catching HIV from him/them during sex. Is/are your partner(s) living with HIV?	*[If client responds “Yes”]* This is important information to share with your provider as you think about your contraceptive choices. You may also wish to discuss with your provider how you can get tested for HIV.
Dual protection	There also is an exciting new medicine that people can take to reduce their risk of getting HIV. It's called pre-exposure prophylaxis or PrEP. This is a daily medication that can help reduce your risk of getting HIV. Some women want to use PrEP in addition to contraceptive methods so they can be protected from both pregnancy and HIV. Would you be interested in learning more about PrEP?	*[If client responds “Yes”]* Great! When you see your provider, this would be a good opportunity to talk about your interest in PrEP, and learn how you can get it.

Abbreviations: FP, family planning; PrEP, pre-exposure prophylaxis; STI, sexually transmitted infection.

After the English draft was written, we conducted 3 in-person testing workshops in Lusaka, Zambia, with Lusaka-based women of different age groups (15–24 years, 25–34 years, and 35–49 years). Workshop participants reviewed each chatbot message to ensure that the wording was comprehensible and engaging and that the flow was logical. After refining the chat content based on the workshop feedback, we translated the content into Bemba and Nyanja. We conducted a second round of workshops with Lusaka women to verify the translations, refining all 3 versions iteratively based on workshop feedback. The finalized content in the 3 test languages was collated in a single Excel workbook for uploading and deployment in a chatbot application.

### Building the Chatbot in Microsoft Azure

Once the chat content was finalized in all 3 languages, we selected the Microsoft Azure Bot Service as the application with which to build the chatbot, taking into consideration its relative simplicity, geographic availability, cost, and available time and human resources. For instance, our chat content was scripted as a series of closed-ended questions and did not require artificial intelligence capabilities; the user’s response to each closed-ended question would lead to a scripted subsequent text or question.

We selected the Microsoft Azure Bot Service as the application with which to build the chatbot, taking into consideration its relative simplicity, geographic availability, cost, and available time and human resources.

We programmed the chat content in the Azure platform in English and iteratively edited it to ensure the sequence of texts was logical and accurate. We repeated this uploading and review process for the Bemba and Nyanja versions of the chat.

**FIGURE 1:**
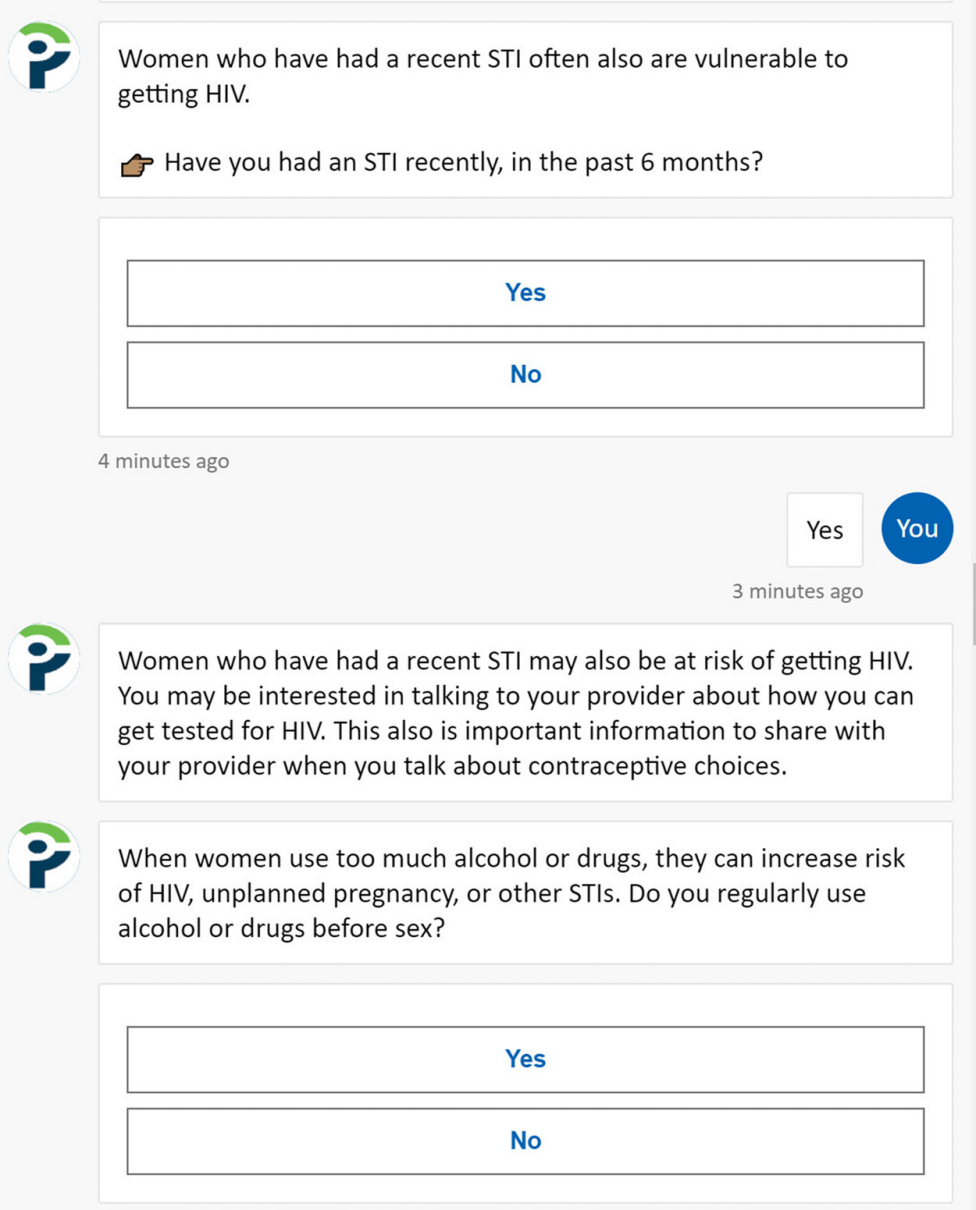
User Interface of HIV/FP Chatbot Abbreviations: FP, family planning; STI, sexually transmitted infection.

Once the back-end programming was complete, we focused on refining the appearance of the front-end user interface of the chatbot, which was accessed on a dedicated web page. For instance, we added occasional simple symbols (Figure 1) and ensured the appearance would be optimized for use on a tablet or other mobile device.

### Ethical Approval

When the content and chatbot development were finalized, we submitted our planned procedures for user testing for ethical review to the Population Council’s institutional review board. The board granted the activity a non-research determination. The aim of the user test was not to produce generalizable knowledge, but to achieve proof of concept and demonstrate the “real world” technological and logistical feasibility of the tool.

## CHATBOT USER TEST

From March 10 to March 23, 2021, we conducted user testing among women in waiting areas of 3 Lusaka public FP facilities: Chawama, Chipata, and Kalingalinga clinics. Consistent with conventional sample sizes employed in user tests of digital tools, we aimed for a sample size of 30–40 users.^18^ Eligible testers were women aged 15 to 49 years who could read Bemba, English, or Nyanja and who possessed sufficient digital literacy to navigate the chatbot on a touch-screen tablet provided by ADVICE staff. To identify potential testers, at each clinic the nurse in-charge approached women in the waiting area and conducted an initial screening for eligible age and reading literacy in 1 of the 3 chatbot languages. Interested women then underwent a secondary literacy screening with an ADVICE staffer, for which she was asked to read several basic questions (such as “Which of the following images shows a banana?” and “Please state how old you are, in years”). Women who passed the literacy screenings were handed an Internet-enabled tablet by the ADVICE staffer, on which the website that hosted the chatbot was preloaded. After specifying a preferred language on the chatbot home page, she then responded to a practice message to ensure her facility with using the device (“Which of the following methods is most effective at preventing pregnancy? [Responses: condom, injectable, pill]”).

After completing the chat, testers completed a 5-minute online exit survey that was self-administered using the same tablet on which they engaged with the chatbot. This survey was completed either before or after seeing the provider. The exit survey solicited their views on using the chatbot and collected their basic demographic information. Testers received a transportation stipend of 60 Zambian kwacha (US$2.68) for participating in the user test.

### Tester Feedback on Chatbot

#### Tester Characteristics

At the 3 facilities, the nurse in-charge and ADVICE staff invited 54 women to test the chatbot, among whom 30 used the chatbot and completed the exit survey. Among the women who declined to participate and/or were determined to be ineligible, 13 did not pass the literacy screening. The other nonparticipants’ reasons for declining to test the tool were that they were “too busy” and “not interested.”

Table 2 presents basic demographic information on the 30 testers. Two-thirds were age 25 years and older, and 80% were married or cohabiting. A majority (80%) had at least secondary education.

**TABLE 2.** Demographic Characteristics of HIV/FP Chatbot Testers in Lusaka, Zambia (N=30)

	No. (%)
Age, years	
<18	1 (3.3)
18–24	9 (30.0)
25–34	19 (63.3)
35+	1 (3.3)
Marital status	
Single, never married	5 (16.7)
Married or cohabitating	24 (80.0)
Divorced/widowed	1 (3.3)
Highest level of education completed	
Primary or less	6 (20.0)
Secondary or more	24 (80.0)

Abbreviation: FP, family planning.

#### Feasibility and Acceptability of the Waiting-Area Chatbot

**FIGURE 2:**
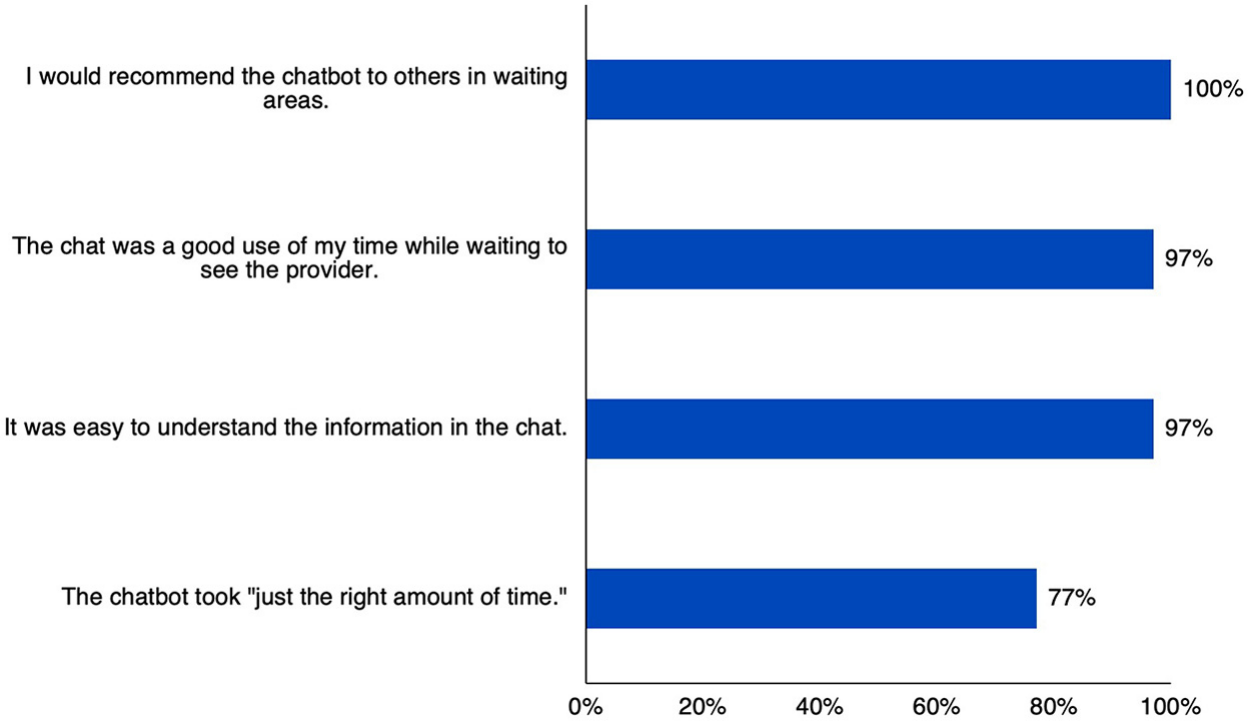
HIV/FP Chatbot User Feasibility and Acceptability (N=30) Abbreviation: FP, family planning.

More than three-quarters of testers felt the chat took “just the right amount of time” to complete, and a large majority felt the tool was easy to understand, a good use of time, and would recommend it to others (Figure 2).

More than three-quarters of testers felt the chat took “just the right amount of time” to complete, and a large majority felt the tool was easy to understand, a good use of time, and would recommend it to others.

In addition, in response to an open-ended question that asked testers if they had any other comments to add, 4 women volunteered that they would like to learn more about PrEP. Others mentioned that they felt the chatbot could benefit other women and girls.

*The chat has helped me to learn a lot, and I would like that other women get this opportunity. —*Married/cohabiting woman, age 25–34 years

*I would like this chat to continue for the good future of young women and girls.* —Married/cohabiting woman, age 18–24 years

One tester noted a limitation of the chatbot.

*[It] could be difficult to navigate for somebody that can't read and understand. —*Married/cohabiting woman, age 25–34 years

Another woman suggested that the ADVICE team should go into the communities to talk about HIV and FP issues.

#### Effect on Knowledge and Provider Interaction

**FIGURE 3:**
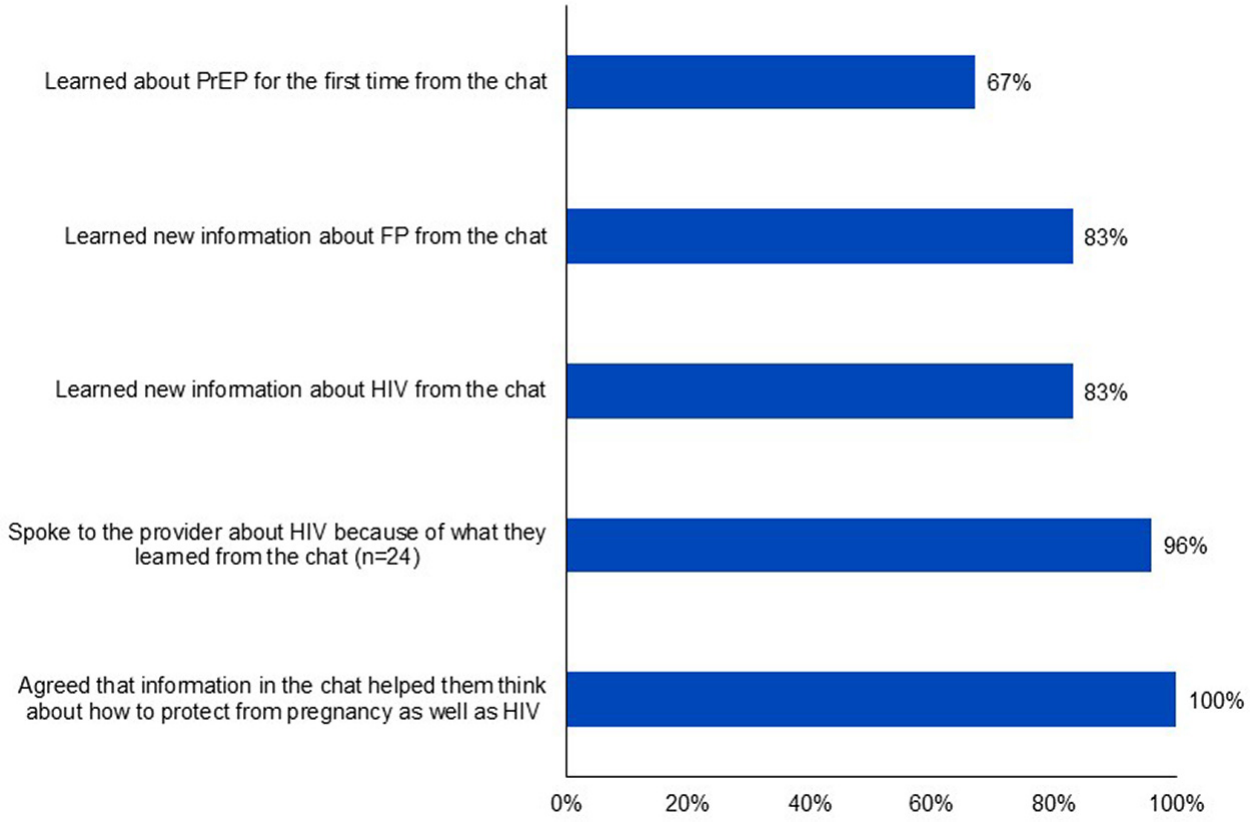
HIV/FP Chatbot Effect on User Knowledge and Provider Interaction (N=30) Abbreviations: FP, family planning; PrEP, pre-exposure prophylaxis.

Twenty-five of the 30 testers (83%) reported learning new information about HIV from the chat, with an equal number stating that they learned new FP information. Twenty (67%) learned about PrEP for the first time through the chat. Out of the 30 testers, 24 completed the exit survey following their consultation with the provider and, thus, were able to report on whether and how their chatbot experience influenced their provider interaction. Nearly all (96%) reported that they spoke to the provider about HIV because of what they learned in the chat. In addition, all agreed that information in the chat helped them think about how to achieve dual protection against both pregnancy and HIV Figure 3).

Most of the testers reported learning new HIV and FP information about HIV from the chat.

### Limitations and Future Opportunities

The purpose of this user test was to achieve proof of concept for a waiting-area chatbot (i.e., demonstrate the viability of this initial prototype), rather than formally piloting or evaluating the tool. Thus, the ADVICE team intentionally removed several barriers to use by, for example, providing users with a tablet, which circumvented the need for testers to have their own devices or use data, and offering testers a transport stipend.

To test the scalability and sustainability of a solution like this chatbot, implementers should explore strategies (e.g., digital marketing, offline educational materials, etc.) that encourage users to proactively engage with the chat on their own mobile devices, in the absence of external facilitators directing users to the digital chat on a facility or project tablet. What are promising strategies for “marketing” the chatbot to users? How can we better circumvent potential connectivity issues? What are provider perspectives on how this tool affects consultations? Future efforts to adapt this innovative intervention should reflect and address these next challenges.

With the ADVICE chatbot content developed and tested, this conversational content (i.e., multilingual text threads collated in an Excel workbook) is now at our disposal to adapt or refine for other contexts and users, beyond the FP/HIV setting. In addition, this content can be used with other digital platforms beyond Microsoft Azure, should the resources, complexity, or time suggest other platforms and/or applications may be feasible and appropriate (e.g., Facebook, WhatsApp, etc.). This chatbot development was subsidized by a substantial amount of human and technological resources leveraged from ongoing activities or contributed in kind, so we are unable to make a meaningful estimate of its cost, nor was the aim of this user test to demonstrate its financial sustainability. Nevertheless, we are encouraged by the broad interest in this concept expressed by the users and potential future funding partners. For instance, we are pleased to report that, in March 2022, the Population Council received support from Gilead Sciences, Inc. to create a revised chatbot to promote PrEP among vulnerable communities in Nigeria, which presents an opportunity to build on the learnings from our user test in Zambia.

This tool was intended to promote self-reflection about HIV among FP clients with basic verbal and digital literacy, which would encourage them to explore any relevant concerns or questions with their providers. Chatbot users’ responses to the digital conversation were not shared with providers, unlike in some U.S. waiting-area FP digital interventions in which providers received, for instance, a print-out of patient preferences to reference during the consultation.^20^^–^^22^ The feasibility and sustainability of such an approach in low-resource settings—which would require dedicated printers and maintenance—are questionable. However, as electronic medical records become more prevalent, it may be possible to link clients’ inputs into a digital waiting-area tool with their medical records, if appropriate and desirable.

## CONCLUSIONS

We not only underwent a rigorous process for developing, testing, and translating the content of the digital conversation (i.e., the HIV/FP information imparted) but also developed end-to-end the front-end interface (i.e., the website with which the user interacts) and the back-end programming via the Azure platform. We describe the steps to develop this innovative product. In addition, we undertook a clinic-based usability test to observe its deployment among potential users. The successful proof of concept of the ADVICE chatbot—both the substance of the content and digital application for its conveyance—demonstrated that preservice, waiting-area time presents an underutilized opportunity to impart HIV information to FP clients, thereby preparing them to discuss their dual HIV and pregnancy prevention needs when they see their providers. By offloading some of the initial counseling burden from the provider, waiting-area information provision can minimize the additional draw on providers’ time, who already are tasked with covering extensive FP content in patient interactions. Furthermore, the testers exhibited notable interest in learning more about PrEP, which underscores the appropriateness and promise of integrating PrEP into FP services.

## Notes

**Peer Reviewed**

**First published online:** September 27, 2022.
